# Overcome cancer drug resistance by targeting epigenetic modifications of centrosome

**DOI:** 10.20517/cdr.2018.010

**Published:** 2019-06-19

**Authors:** Zan-Hui Jia, Xing-Gang Wang, Hong Zhang

**Affiliations:** ^1^Second Hospital of Jilin University, Changchun 130000, Jilin Province, China.; ^2^Department of Biomedicine (DBM), University Hospital, University of Basel, Basel 4001, Switzerland.

**Keywords:** Centrosome, genomic instability, epigenetic disturbance, carcinogenesis, cancer, clinical trials

## Abstract

The centrosome is an organelle that serves as the microtubule- and actin-organizing center of human cells. Although the centrosome is small of size, it is great important on cellular function that regulates cytoskeletal organization and governs precise spindle orientation/positioning ensuring equal distribution of cellular components in cell division. Epigenetic modifications to centrosome proteins can lead to centrosome aberrations, such as disorganized spindles and centrosome amplification causing aneuploidy and genomic instability. Epigenetic disturbances are associated not only with carcinogenesis and cancer progression, but also with drug resistance to chemotherapy. In this review, we discuss mechanisms of epigenetic alteration during the centrosome biogenesis in cancer. We provide an update on the current status of clinical trials that aim to target epigenetic modifications in centrosome aberrations and to thwart drug resistance.

## Introduction

It has long been considered that the accumulation of genetic mutations in tumor suppressors and/or oncogenes causes cancer^[1]^. However, mounting evidences have emerged that alterations of every component in the epigenetic regulatory machinery also participate in carcinogenesis^[2,3]^. Ultimately both genetic and epigenetic changes determine abnormal gene expression. Centrosomes play a key role in establishment and maintenance of the bipolar mitotic spindle that require to accurately divide genetic material (chromosomes) into daughter cells during cell division. Centrosome aberrations are either numerical or structural aberrations since they arise when centrosome structure, duplication or segregation are deregulated. So far, it has not been shown whether structural centrosome aberrations directly trigger drug resistance. In this review, we only focus on numerical aberrations of centrosome. Acquisition of ≥ 3 centrosomes in the centrosome cycle was termed centrosome amplification. Failure to properly control centrosome number leads to aneuploidy, which is frequently found in cancer cells. Mechanistically centrosome amplification may cause multipolar spindles or monopolar aster resulting in chromosome missegregation^[4]^. Thus, centrosome amplification is a hallmark of human tumors^[5-10]^. The BRCA1 E3 ligase specifically ubiquitinated γ-tubulin at lysine-48 (K48) and the expression of a mutant γ-tubulin protein in which K48 was mutated to arginine induces centrosome amplification^[11]^. However, centrosome amplification does not necessarily require DNA damages, epigenetic changes is one potential mechanistic link in dysregulation of centrosome function. Here we discuss in detail on centrosome structure, aberrations of centrosome in cancer, the relationship of cancer drug resistance to centrosome amplification and new drug development.

## Centrosome aberrations, cancer and cancer drug resistance

**Figure 1:**
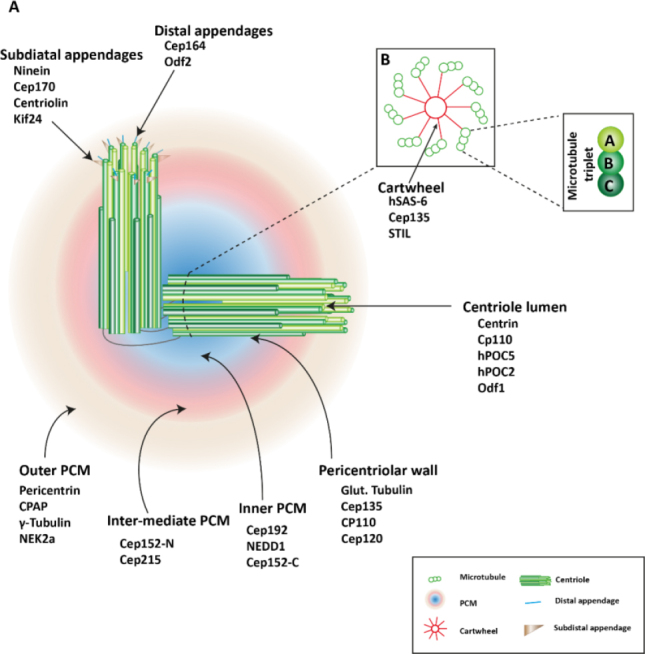
Centrosome structure. A: the centrosome consists a pair of orthogonal centrioles, surrounded by mass proteins, referred to as the pericentriolar material (PCM). PCM proteins subdivide the PCM to three layers depending on diameter of the ring-like structures. Mother centrioles possess subdistal appendages and distal appendages; B: each centriole is a cylindrical structure with nine-fold microtubule organized like a central cartwheel. The triplet microtubules are composed of internal A, middle B, and external C microtubules. The figure is adapted and modified^[9]^

**Figure 2:**
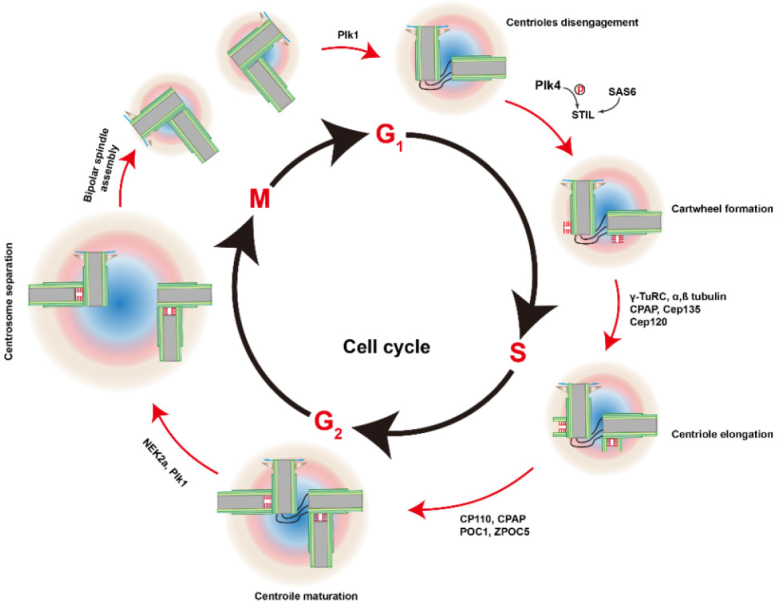
Centrosome duplication cycle. Centrosome duplication begins at the G1-S transition, with the disengagement of the pair of centrioles. Plk4 binds and phosphorylates STIL and associates with SAS-6. Thus, the cartwheel forms the proximal wall of the mother centriole. Other proteins are recruited to the cartwheel and the new daughter centriole (procentriole) begins to grow from the existing centrioles during S and reaches the full length at the G2 phase. Duplicated centrosomes separate at the beginning of the M phase, helped by kinases NEK2a and Plk1. PCM mature and the cartwheels disassemble in the early M phase. After the separation of two daughter cells, each cell inherits one centrosome. The figure is adapted and modified^[15]^

In this section, we briefly summarize the structure and biogenesis of centrosomes (for more detail reviews, please refer to^[12]^. Morphologically, centrosomes are non-membranous organelles. Each centrosome consists of a pair of centrioles surrounded by the pericentriolar material (PCM) [Figure 1]. Although molecular compositions of the centrosome remain poorly defined, hundreds of proteins have been detected using proteomics^[13,14]^. As illustrated in Figure 2^[15]^, cycling cells begin the cell cycle with one centrosome in G1, and centriole duplication occurs once per cell cycle, paralleled with DNA replication during the S phase. The duplication of the centrosome is initiated (formation of the procentriole) with Plk4 as the dominant kinase in centriole biogenesis^[16,17]^. Depletion of Plk4 induces the loss of centrioles and overexpression of Plk4 conversely causes the formation of multi-daughter centrioles^[18]^.

Once centrioles are assembled, PCM proteins will be recruited, nucleating more microtubules during interphase. These proteins are not only important for centrosome biogenesis but also contribute to the maintenance of cell polarity, cell vesicle transport, cell adhesion, cell signal transduction (Reviewed^[19]^). Duplicated centrosomes separate at the onset of mitosis for bipolar spindle formation, which equally segregate sister chromatids to two daughter cells. Centrosome disjunction is modulated by NEK2a to remove centrosomal linkers, such as C-Nap1 (also known as CEP250) and rootletin^[20]^). Over-expression of NEK2a induces immature centrosome separation^[21,22]^.

It is worth to note that majority of centrosome proteins have multiple locations. Approximately 77% (*n =* 370) of the centrosome and microtubule-organizing center (MTOC) proteins detected in the cell atlas also localize to other cellular compartments^[23]^. The network plot shows that the most common locations shared with centrosome and MTOC are the cytoplasm, nucleus and vesicles. For example, CTCF is associated with the centrosome in metaphase to anaphase of the cell cycle. At telophase, CTCF dissociates from the centrosome and localizes to the midbody and the newly formed nuclei.

Functionally the centrosome in human cells acts as the MTOC, which has been studied widely ever since first described by Theodor Bovery in 1900^[24]^, and the actin-organizing center^[25]^. Importantly precisely duplicated and matured centrosomes ensure faithful chromosome segregation into two daughter cells via the formation of the bipolar mitotic spindle^[26]^. Thus, when centrosome structure, duplication or segregation are deregulated, centrosome aberrations with either numerical or structural aberrations arise.

Many studies established a link of centrosome aberrations and solid tumors or hematological malignancies; the correlations are present not only in pre-invasive lesions but also with tumor progression^[27,28]^. Although bipolar spindles could be detected in centrosome depletion or centrosome over-duplicated cells by clustering mechanisms^[29]^, the numerical centrosome aberrations are still the most common cause for chromosomal segregation errors^[30]^. Centrosome amplification thus can be as a novel biomarker for personalized treatment of cancers^[31]^. Several oncogenic and tumor suppressor proteins, such as BRCA1 and p53, are the best-known centrosome proteins^[32,33]^. Either overexpression or downregulation of centrosome proteins evoke centrosome abnormalities resulting tumorigenesis [Table 1].

**Table 1:** Association of centrosome proteins with epigenetic disturbances in different types of tumors

Cancer marker	Cancer types	Ref.
USP9X	Breast	[34]
BRCA1	Breast	[32]
p53	Breast	[33]
Cep70	Pancreatic	[35]
Pericentrin	Breast, bladder	[36,37]
Nek2	Prostate, breast, melanoma	[38-40]
TACC3	Gastric, melanoma, ovarian	[41-43]
Plk4	Breast, melanoma	[44,45]
Aurora A	Esophageal	[46]
CTCF	Breast	[47]

As discussed above, NEK2 is an important centrosome protein, which regulates centrosome separation and bipolar spindle formation in mitotic cells. High expression of NEK2 also mediates drug resistance to cisplatin or lipo-doxorubicin in myeloma^[48]^, breast cancer^[49]^, ovarian cancer^[50]^ and liver cancer^[51]^. Nlp (ninein-like protein) is involved in centrosome maturation and spindle formation. Nlp overexpression was detected in human breast and lung cancers^[52]^. By examining 55 breast cancer samples, a study found that the breast cancer patients with high expression of Nlp were likely resistant to the treatment of paclitaxel. KIFC1 is a nonessential minus end-directed motor of the kinesin-14 family and it functions as a centrosome clustering molecule^[53,54]^. In breast cancer cells, overexpression of KIFC1 and KIFC3 confer docetaxel resistance^[55]^. These studies indicate that centrosome aberrations not only produce aneuploidy, chromosome instability leading to tumorigenesis but also promote cancer drug resistance.

## Mechanisms of cancer drug resistance involving centrosome

The mechanism underlying chemo-resistance (mitotic drug resistance) is not yet clear. Several studies provide insights into the molecular basis of centrosome abnormalities that produce drug resistance, either directly by dysregulation of centrosome protein levels or indirectly by regulating gene expression of other proteins.

### Gene dosage

As discussed previously, gene dosage may affect centrosome duplication through a balance of the relative abundance of one or more proteins essential for the assembly of new centrioles and the availability of assembly sites. Thus, dysregulation of gene dosage for centrosome proteins produce aneuploidy and chromosome instability. Moreover, there are a close relationship between aneuploidy, chromosome instability and chemotherapy resistance.

The Aurora A kinase regulates centrosome maturation and separation and thereby play important roles in spindle assembly and stability. Overexpression of Aurora-A kinase induces centrosome amplification and chromosomal instability that create tumor cell heterogeneity, thus is associated with acquired drug resistance^[56]^.

PLK4 is a key component of the centrosome. Dysregulation of PLK4 activity causes loss of centrosome numeral integrity. Its overexpression is responsible centrosome amplification and contributes to resistance to tamoxifen and trastuzumab^[57]^.

### Mitotic slippage

Chemotherapy is commonly used in order to induce cell death or to prevent proliferation of cancer cells by impairing spindle function and chromosome segregation. However sometimes cancer cells evade cell death for those that are arrested in mitosis^[58]^. Instead these cells leave mitosis without completing a normal cell division and become tetraploid. This phenomenon is called mitotic slippage. The examples can be seen in the drugs that target microtubule assembly (nocodazole, vincristine) or the disassembly (taxol or paclitaxel).

Avoiding apoptosis through mitotic slippage in cancer cells is thought to be a major mechanism contributing to cancer drug resistance. An interesting recent study provides insight into the mechanism of mitotic slippage. BH3-only pro-apoptotic proteins are necessary to initiate the molecular process of apoptosis in cells undergoing perturbed mitosis^[59]^. NEK2 conferred drug resistance is associated with decreased apoptosis^[60]^. Furthermore, overexpression of NEK2 suppressed the expression of the BH3-only genes BAD and PUMA and upregulated the expression of pro-survival genes BCL-xL and MCL-1, indicating a possible role of NEK2 in cancer drug resistance via mitotic slippage.

Phosphorylation at S69 of BIM, which is also a BH3-only protein, leads to its ubiquitin-dependent degradation^[61]^. During mitotic arrest, BIM is known to be heavily by Aurora A kinase, which could result in mitotic slippage.

Centrosome protein CEP55 was found to have a role in promoting mitotic slippage, which again is mediated by the Bcl2 family proteins in breast cancer^[62]^. In breast cancer patients, high-level expression of CEP55 associates with chemotherapeutic resistance, particularly to docetaxel. Similarly, docetaxel induces spindle multipolarity, higher KIFC1 expression might counteract this effect to prevent cell death and enable bipolar spindle formation through centrosome clustering^[55]^.

These studies demonstrated that centrosome proteins regulate gene expression of apoptotic/anti-apoptotic genes to induce cancer drug resistance.

### Regulation of drug transporters by centrosome proteins

A recent study demonstrates that overexpression of NEK2 and drug resistance are closely correlated in other cancers through activation of efflux drug pumps^[57,60]^. Overexpression of NEK2 upregulated ABC transporter family members, including ABCB1 (p-glycoprotein, MDR1), the multidrug resistance protein ABCC1 (MRP1), and the breast cancer resistant protein ABCG2 (BCRP). High expression of NEK2 promoted a higher efflux of the hydrophilic eFluxx-ID gold fluorescent dye from cancer cells. Verapamil, an ABC transporter inhibitor, was able to abrogate part of the NEK2-induced drug resistance by showing a decrease in colony formation. Downregulation of NEK2 by shRNA decreased the expression of phosphorylated PP1, AKT, nuclear β-catenin, and ABC transporters.

However, it is also worth to note that drug resistance can also be secondary since chemotherapy or radiation may induced centrosome abnormalities. This is associated with tumor cell heterogeneity. Accumulations of centrosome aberrations after nilotinib and imatinib treatment in vitro are associated with mitotic spindle defects and genetic instability^[63]^.

## Epigenetic regulation of centrosome protein expression

Epigenetic mechanisms that alter functional gene dosage through hyper- or hypo-methylation, and consequently the abundance of key centrosome precursor molecules, may result in centrosome abnormalities, spindle defects, aneuploidy and polyploidy.

Although any genetic aberrations of the centrosome proteins contributes to tumorigenesis, alterations of epigenetic gene regulation are found more frequently as cancer drivers, which include widespread alterations of CpG island methylation, histone modifications, and dysregulation DNA binding proteins disrupt normal patterns of gene expression.

### Phosphorylation

Many kinases (e.g., CDKs, Aurora A, polo-like kinases, *etc*.) participate in the regulation of centrosome duplication even they themselves are also controlled by phosphorylation and dephosphorylation. For instance, the phosphorylation status of CKAP2 during mitosis is critical for controlling both centrosome biogenesis and bipolar spindle formation^[64]^. It has been shown that the Cyclin-Dependent Kinase (CDK)-activating phosphatase (CDC25B) localizes to centrosome and involves in the centrosome duplication cycle and in microtubule nucleation^[65]^. The activity of CDC25B is positively or negatively regulated by several kinases including Aurora A and CHK1^[66,67]^. The phosphorylation of CDC25B by Aurora-A locally participate in the control of the onset of mitosis^[68]^. Abnormal expression of CDC25B in numerous human tumors might have a critical role in centrosome amplification and genomic instability^[69]^.

Activities of Aurora-A (AurA) for its cellular function are regulated by different protein-protein interactions and posttranslational modifications. It has been established that Twist1 has a critical role in promoting EMT and drug resistance. AURKA phosphorylates Twist1 at three positions (S123, T148 and S184). AURKA-mediated phosphorylation of Twist1 is crucial for EMT, the cancer stem cell phenotype and drug resistance togemcitabine^[70]^. On the other hands, activation of AurA at centrosomes occurs through autophosphorylation at the critical activating residue Thr288^[71]^. The autophosphorylation is regulated by PLK1^[72]^ and TPX2^[73]^.

PLK4 contains an N-terminal kinase domain (residues 12-284) a C-terminal localization domain (residues 596-898) and 3 polo box domains, which facilitates oligomerization, targeting, and promotes trans-autophosphorylation. PLK4 can be directly phosphorylated and activated by stress-activated protein kinase kinase kinases^[74]^. Indeed, tumor-derived SAPKK1/MKK4 mutants induced centrosome amplification under genotoxic stress (only in p53-negative cells)^[74]^, which lead to increased resistance to apoptosis, chemotherapy and radiotherapy^[75]^.

PLK4 is also a substrate of itself (via autophosphorylation). Autophosphorylation of PLK4 results in ubiquitination and subsequent destruction by the proteasome^[76-78]^. It has been shown that mutagenesis of ASP-154 in the catalytic domain that causes centrosome amplification above background levels when overexpressed^[16]^. This may be due to the loss of self-destruction of PLK4.

### Acetylation

Acetylation and deacetylation are highly common posttranslational modifications. Several studies demonstrate that acetylation/deacetylation play a role in the regulation of centrosome duplication and induction of abnormal amplification of centrosomes. KAT2A/KAT2B function as histone acetyltransferase or lysine acetyltransferases. Fournier and her colleagues showed that KAT2A/2B acetylate the PLK4 kinase domain on residues K45 and K46^[79]^. Impairing KAT2A/2B-acetyltransferase activity results in diminished phosphorylation of PLK4 and in excess centrosome numbers in cells. Therefore KAT2A/2B acetylation of PLK4 prevents centrosome amplification. On the other hands, through focusing on the deacetylases, Fukasawa’s group found that the deacetylation event negatively controls centrosome duplication and amplification. Of the 18 total known deacetylases (HDAC1-11, SIRT1-7), ten deacetylases possess the activity to suppress centrosome amplification, and their centrosome amplification suppressing activities are strongly associated with their abilities to localize to centrosomes. Among them, HDAC1, HDAC5 and SIRT1 show the highest suppressing activities, but each of them suppresses centrosome duplication and/or amplification with its unique mechanism^[80]^.

### Methylation

G9a is a histone methyltransferase enzyme, also known as euchromatic histone-lysine N-methyltransferase 2 (EHMT2)^[81]^. G9a catalyzes the mono- and di-methylated states of histone H3 at lysine residue 9 (i.e., H3K9me1 and H3K9me2) and lysine residue 27 (H3K27me1 and HeK27me2). G9a plays a critical role in regulating centrosome duplication. Knockdown of G9a significantly reduces di- and trimethylation of H3K9, resulting in disruptions in centrosome amplification and chromosome instability in cancer cells^[82]^. Furthermore, silencing G9a leads to down-modulation of gene expressions, including that of p16INK4A. It has been shown that cells lacking p16 (INK4A) activity exhibit phenotypes associated with malignancy^[83]^. p16INK4A is the CDK2, Cdk4 and Cdk6-specific inhibitor^[84]^. The observations of the effects on G9a silencing are in support of the studies linking cyclin D1/Cdk4 with centrosome amplification^[85,86]^. Initiation of tumorigenesis was found in the loss of p16INK4A through hypermethylation of its promoter^[87-89]^. Thus, it has been postulated that loss of p16 expression coupled with increased γ-tubulin contributes to centrosome amplification and breast cancer progression^[90]^.

14-3-3 proteins are associated with centrosomes^[91]^. 14-3-3γ prevents centrosome amplification and neoplastic progression^[92]^. Inactivation of the 14-3-3 sigma gene is associated with 5’ CpG island hypermethylation in human cancers^[93,94]^. Promoter hypermethylation of p53 genes is detected in many cancers^[95-97]^.

### Promoter

There is a functional link between centrosome and transcription factors. NF-κB can induce abnormal centrosome amplification by upregulation of CDK2^[98]^. A functional NF-κB binding site was located in the CDK2 promoter.

Methyl-CpG binding protein 2 (MeCP2) localizes at the centrosome. Its loss causes deficient spindle morphology and microtubule nucleation. In addition, MECP2 binds to histone deacetylases and represses gene transcription^[99]^.

E2Fs affect the expression of proteins, including Nek2 and Plk4, thereby deregulation of E2Fs induces centrosome amplification in breast cancer^[100]^. A further example showed that arsenic induced centrosome amplification via SUV39H2-mediated epigenetic modification of E2F1^[101]^.

DDX3 regulates epigenetic transcriptional and translational activation of p53 and colocalizes with p53 at centrosome during mitosis to ensure proper mitotic progression and genome stability, which supports the tumor-suppressive role of DDX3^[102]^. DDX3 knockdown suppressed p53 transcription through activation of DNA methyltransferases along with hypermethylation of p53 promoter and promoting the binding of repressive histone marks to p53 promoter.

During tumor development, especially to most solid tumors, cancer cells are often subjected to hypoxia^[103]^. A recent study showed that via upregulation of HIF1, proteins whose overexpression drives centrosome amplification (such as Cyclin E, Aurora A, and PLK4) were upregulated^[104]^.

### MiRNA

Recently gene expression of centrosome proteins was found as miRNA targets. MiR-129-3p is identified as a novel metastatic microRNA. CP110 expression was repressed by miR-129-3p^[105]^.

PLK1 is one of targets of miRNA-210-3p, and lnc-RI regulates PLK1 mRNA stability by competing with the PLK1 mRNA 3’UTR for binding to miRNA-210-3p^[106]^.

MiR-128 inhibited NEK2 expression and miR-128 was silenced by DNA methylation. Up-regulation of NEK2 by MicroRNA-128 methylation is associated with poor prognosis in colorectal cancer^[107]^.

### Ubiquitination

Dysfunction in the ubiquitin-proteasome degradation has implicated in several cancer drug resistance. MDM2 is an E3 ubiquitin-protein ligase that mediates ubiquitination and degradation of p53^[108,109]^. Increased levels of MDM2 would inactivate the functions of p53 to similar extent that do in deletion or mutation of p53 and found in a variety of human tumors^[110]^. Several studies demonstrated that MDM2 overexpression increases cancer drug resistance of tumors^[111,112]^. Mind bomb (Mib1) was identified as the E3 ubiquitin ligase of PLK4^[113]^. Recently we found that HECTD1, a HECT-type E3 ubiquitin ligase is a novel centrosome protein whose deficiency induces centrosome amplification and promotes epithelial-mesenchymale transition^[114,115]^. These results indicate ubiquitination is one of the important epigenetic modifications for centrosome.

## New drugs in trial

Abnormalities in size, number and microtubule nucleation capacity of centrosome are resulted from genetic disorders or epigenetic disturbances of gene expression. Epigenetic modifications are temporally dynamic and reversible changes. Development of small molecules targeting epigenetic regulators are promising anticancer strategies, involving elimination of cancer cells with chromosome instability and aneuploid in combination with targeting centrosome proteins to overcome mitotic slippage and to induce apoptosis in cancer cells. The drugs that focused on the centrosome amplification may provide possibilities to treat cancer or overcome some forms of drug resistance. Recently clinical trials of inhibitors targeting kinases that function as centrosome regulators are under way for hematologic malignancies and solid tumors. We summarize the development of therapies targeting these mechanisms.

Although a lot of progresses have been made in treating cancer, the most cancer chemotherapeutics develop drug resistant (secondary) that limits the efficacy of treatments. This happened even for the newly approved NTRK inhibitors^[116]^. Thus, there is a significant need to target drug resistance for improved therapeutics for cancer. Several clinical trials, in single or combination of drugs, have been focused on blocking centrosome clustering to combat drug resistance [Table 2].

**Table 2:** Ongoing trials with known inhibitory activity to centrosome aberrations

NCT number	Start date	Drug	Targets	Cancers	Phases	Patient number
NCT03654716	01 2018	ALRN-6924	MDM2 and MDMX	Solid tumor	1	69
NCT03634228	08 2018	DS-3032b	MDM2	Refractory Acute Myeloid Leukemia	2	52
NCT02098967	03 2014	RO6839921	MDM2	Neoplasms, Myelogenous Leukemia, Acute	1	68
NCT03671564	09 2018	Milademetan	MDM2	Acute Myeloid Leukemia	1	24
NCT03787602	12 2018	KRT-232	MDM2	Merkel Cell Carcinoma	2	27
NCT03781986	03 2019	APG-115	MDM2	Malignant Salivary Gland Cancer	2	62
NCT03566485	06 2018	Atezolizumab (an Anti-PD-L1 Monoclonal Antibody) With Idasanutlin	PD-L1/MDM2	Metastatic ER + Breast Cancer	1+2	92
NCT01014429	11 2009	NMS-1286937	PLK1	Advanced/Metastatic Solid Tumors	1	21
NCT01954316	10 2013	CFI-400945	PLK4	Advanced Cancer	1	48
NCT02187783	11 2014	LEE011	CDK4/6 Pathway	CDK4/6 Pathway Activated Tumors	2	106
NCT03242382	08 2017	Palbociclib	CDK4 Overexpression	Advanced Sarcomas	2	38
NCT00536835	09 2007	GSK461364	PLK1	Non-Hodgkins Lymphoma	1	40
NCT03555877	06 2018	Ribociclib	CDK4/6	Breast Cancer Metastatic	2	150
NCT01676753	06 2016	Dinacicli + pembrolizumab	Cyclin-dependent Kinase	advanced breast cance	1	32
NCT01037790	2009	PD-0332991	CDK4 mutation	Solid Tumor	2	205
NCT03242382	08 2017	Palbociclib	CDK4 Overexpression	Sarcoma	2	38
NCT03024489	01 2017	Palbociclib + cetuximab	CDK4 Overexpression	Head and Neck Cancer	2	33
NCT03310879	11 2017	Abemaciclib	Abnormality in one of the following genes: CCND1, CCND2, CCND3, CDK4, or CDK6	Breast	2	38
NCT03050398	06 2017	Ribociclib+ letrozole	CDK4/6	breast cancer	3	140
NCT03096912	06 2016	Ribociclib	CDK4/6	Liposarcoma	2	30
NCT03054363	11 2017	Palbociclib + letrozole	CDK4 overexpression	Hormone Receptor Positive and HER2-positive Metastatic Breast Cancer	1 + 2	40

NCT: National Clinical Trial

Since increased levels of MDM2 inactivate the functions of p53 thereby induce centrosome amplification and drug resistance. Inhibition of MDM2 is obviously a good strategy to fight cancer drug resistance. Several clinical trials have been setting up to a variety of human tumors, such as Leukemia, Myeloma, Brain Tumor, Solid Tumor and Lymphoma [Table 2].

As mentioned above, PLK4 is important in centrosome biogenesis and regulates mitotic progression. PLK4 has, therefore been identified as a candidate anticancer target. With directed virtual screening using a ligand-based focused library, several leads have been identified. CFI-400945 was generated through further optimization^[117]^. CFI-400945 is a potent and selective small molecule inhibitor of PLK4^[118]^.

Monopolar spindle 1 (Mps1/TTK) kinase is essential for safeguarding proper chromosome alignment and segregation during mitosis^[119]^. Its overexpression contributes to more aggressive and drug resistant breast tumors but the reduction of the Mps1 level can sensitize several tumor cells to paclitaxel^[120]^. In the two ongoing clinical trials, the Mps1 inhibitors are tested along with paclitaxel in triple negative breast cancer patients [Table 2, NCT02138812 and NCT03328494].

Plk1, known as polo-like kinase 1, supports the functional maturation of the centrosome and establishment of the bipolar spindle. Overexpression of Plk1 is often observed in cancer cells^[121]^. This protein therefore is a potential drug target in cancer^[122]^. Several inhibitors of PLK1 have been developed and the promising results have been obtained in clinical trials^[123]^. For example, as compared to administration of cytarabine (a chemotherapy medication used to treat acute myeloid leukemia ) alone, a combination of BI 6727 with cytaribine increased the response with 31% total remission from 13%^[124]^.

Cyclin-dependent kinases (Cdks) are a family of protein kinases that regulate the centrosome cycle but deregulation of those Cdks by oncogenes and tumor supressors results to centrosome amplification^[125]^. More than 30 small-molecule inhibitors developed^[126]^. Many of them have been used in clinical trials studies for the treatment of various cancers [Table 2]^[127]^. Flavopiridol is the first Cdk inhibitor used in clinical trials^[128]^. Flavopiridol has been successful in the treatment of AML and chronic lymphocytic leukemia^[129,130]^.

The Aurora kinases (AURKs) are involved in different aspects of mitotic control during cell cycle. Importantly, PLK1 is activated by AURKA/B. Therefore AURKs are potential targets against centrosome for cancer therapy. More than 30 AURK inhibitors have been developed and used in clinical studies^[131]^. For example, the inhibitor MLN8237 (alisertib), which targets AURKA, showed promising efficacy in several solid tumors^[132]^. AZD1152 (barasertib) is a selective inhibitor of AURKB and has been effective in AML patients with an overall response rate of 25%, but with no effective results in patients with solid tumors. In addition, AURKB/AURKC kinase inhibitor GSK1070916A is actually being tested in patients with solid tumors and phase I in the clinical trial has been completed.

## Conclusion

The consequences of numerical aberrations/centrosome amplification leading to tumorigenesis have been studied extensively. In contrast, studies on mechanisms of cancer drug resistance in relation to centrosome aberrations have received little attention. Epigenetic modifications in centrosome biogenesis have important implications for the origin of some malignant tumors and play a role in cancer drug resistance. The current review discussed the connection of epigenetic changes causing centrosome aberrations to cancer drug resistance. For clinical, the ultimate goal is to identify effective cancer therapy. So far, most of clinical trials targeting possible drug resistance, which were registered to ClinicalTrials.gov at NIH are still monotherapies and in early stages of development. One important factor we should not forget is that regulation pathways to epigenetic modifications of centrosome, such as positive or negative feedback signaling circuits involved in cancer drug resistance are more complex than once thought. The selection of drugs, together with other treatment like immunotherapy, for combination therapy may lead to improve efficacy and to thwart drug resistance. In the future, one need to address molecular mechanisms how the trafficking of centrosome proteins between centrosome and nucleus determine expression/subcellular localization of downstream signaling molecules, such as the Bcl2 family proteins and ABC transporters. Further understanding of centrosome biology including basic cell biology or pathobiology of epigenetic controls in centrosome will provide potential to establish translatable strategies for cancer treatment and to prevent drug resistance.

## Declarations

### Copyright

© The Author(s) 2019.
